# “The Logic of Care” – Parents’ perceptions of the educational process when a child is newly diagnosed with type 1 diabetes

**DOI:** 10.1186/1471-2431-12-165

**Published:** 2012-10-20

**Authors:** Lisbeth Jönsson, Inger Hallström, Anita Lundqvist

**Affiliations:** 1 Department of Health Sciences, Lund University, Lund, Sweden

**Keywords:** Diabetes education, Children, Parents, Qualitative research, Type 1 diabetes

## Abstract

**Background:**

The number of new cases of type 1 diabetes mellitus (T1DM) has increased substantially in recent years and it is now one of the most common long-term endocrine disorders in childhood. In Sweden the child and family are hospitalised in accordance with the national guidelines for one to two weeks at diagnosis. The purpose of this study was to describe parents’ perceptions of the educational process when their child is newly diagnosed with T1DM.

**Methods:**

Qualitative interviews were performed in the south western part of Sweden with ten mothers and eight fathers of children, diagnosed with T1DM, at three to six months after they had received the diagnosis. The interviews were analysed using deductive content analysis and Mol’s philosophical theory.

**Results:**

The results show that almost all parents had experienced the educational process as being satisfactory. However, most parents felt that the teaching needed to be adapted to the individual families and to help them to learn to live with diabetes in their everyday lives. Rather than merely teaching according to a fixed schedule and cramming knowledge, the education should be parent-centered and provide time for grief and shock. There should also be a greater emphasis on why certain things should be done rather than on what should be done. The routines learned at the hospital made the efforts to be good parents managing the child’s disease, and continuing to lead a normal family life, a difficult task.

**Conclusions:**

In order to optimize the educational process for families with children newly diagnosed with T1DM an increased focus on the families’ perceptions might be helpful in that this could lead to further revelations of the educational process thus making it more understandable for the family members involved.

## Background

More than 346 million people worldwide have diabetes [1] and the number of new cases of childhood-onset type 1 diabetes mellitus (T1DM) has increased substantially in recent years, particularly among younger children [2]. In Europe about 94,000 children had been diagnosed with T1DM until 2005, and it is predicted that in 2020 there will be approximately 160,000 cases [3]. When children are diagnosed with T1DM it is the parents who need to take the responsibility for the daily management of the child’s disease. In spite of this, it is also necessary for the children, depending on their age, to have an understanding of the conditions of diabetes and of how to manage them [4]. Therefore, it is important that patients and parents are actively involved in the care [5]. The aim of diabetes care is to maintain normal blood glucose levels so as to allow for the normal growth and development of the child [6]. However, given the multiple medical and behavioural demands inherent in the contemporary management of diabetes, such as the checking of blood glucose levels, the administration of insulin injections and the control of eating habits and activities, it is evident that many parents experience a range of emotional responses with anxiety being the most consistent symptom [7]. Thus, it is important that professionals are aware of the parents’ vulnerability at the time of diagnosis [8].

The educational process starts immediately after the child is diagnosed with T1DM. Most often a multi-professional team, with special knowledge of children consisting of a diabetes specialist paediatrician (DSP), a paediatric diabetes specialist nurse (PDSN), a dietician, a social worker and a psychologist [9], is responsible for the educational process. The Swedish educational process is based on national guidelines [10] and follows the recommendations of the International Society for Paediatric and Adolescent Diabetes (ISPAD) with regard to the management of T1DM in children and young people [9]. This includes a checklist which, in detail, guides the formal content of the knowledge and skills that the family needs in order to be able to cope [11]. In Sweden the teaching of the parents and child about the management of the child’s diabetes takes place in hospital for a period of one to two weeks. The family’s ability to manage the self-care is tested by granting them leave from the hospital; first for some hours and then for a day and a night before discharge [12]. The purpose of this study was to describe parents’ perceptions of the educational process when their child is newly diagnosed with T1DM.

## Methods

### Study design

A qualitative descriptive method was used to achieve a deeper understanding of the educational process during the initial stage after a child is diagnosed with T1DM. The data were collected through interviews which were analysed with a deductive content analysis [13,14].

### Setting

The study was conducted in the south western part of Sweden and included three paediatric departments. Each paediatric department cared for about 20–30 children, newly diagnosed for T1DM each year. The number of children from 0–18 years in the catchment area of each department was about 55,000-70,000. At each department the diabetes team consisted of a DSP, a PDSN, a dietician, a social worker and a psychologist. The DSP and the PDSN have the main contacts with the child and the family, the dietician has appointments with the families on a regular basis and the psychologist and social worker have appointments on demand during the hospital stay.

### Participants

Inclusion criteria for participants were that they were parents of children aged 3 to 16 years who had been diagnosed with T1DM. Consecutively fourteen families were asked to participate during a six month period and 10 agreed to participate. The PDSN at each hospital who recruited the parents contacted them and gave them both verbal and written information about the study as well as a form for informed consent. Parents who agreed to participate sent the signed informed consent to the PDSN. Subsequently the informed consents were handed over to the first author (LJ) who in turn contacted the families. The interviews were conducted between the spring and autumn of 2010.

A total of 18 individuals were interviewed, including 16 cohabiting parents, one mother not living with the father of the child, and one single mother. The families came from both rural and urban areas. Demographics of the participating parents and their families are shown in Table 1.

**Table 1:** Demographic characteristics of parents and family (N=18)

**Characteristic**	**n**
*Age*	
31-35	2
36-40	15
41-45	0
46-	1
*Gender*	
Female	10
Male	8
*Marital status*	
Married	13
Cohabitation	4
Single	1
*Education*	
Elementary school	0
Secondary school	7
University	11
*Child’s age at diagnosis*	
3-6	3
7-10	3
11-14	2
14-	2
*Siblings*	
0	1
1-2	8
3-	1
*Experience of type 1 diabetes in the family*	
Yes	3
No	15
*Place to live*	
Private house	17
Flat	1

The study was conducted according to the Helsinki declaration [15]; including information about the purpose of the study, time commitment, confidentiality, the voluntariness, and the right to discontinue participation at any time; also, informed written consent was obtained. The study was approved by the Research Ethics Committee of the Medical Faculty, Lund University, Sweden (2007/305, 2009/371). Permission was obtained from the chief physician at all departments involved.

### Conducting the interviews

The first (LJ) and the third authors (AL) interviewed the parents at the same time but in separate rooms. The authors alternated between interviewing mothers and fathers. The interviewers had no previous experience of working with children diagnosed with T1DM, but extensive experience of working with children and families in paediatric care. Before the interview started the parents were informed that all data were handled confidential and that nothing from the interview would be passed on to the staff at the hospitals. The interviews started with an open-ended request: “Please narrate your experiences of the care during hospitalisation when your child was diagnosed with type 1 diabetes”. An interview guide was developed, based on the results of interviews among paediatric diabetes teams, and focusing on the initial educational process [12]. The guide included six areas (Table 2), which were used in cases in which these subjects were not brought up by the parents in the interview. During all the interviews, probing questions for clarification – e.g. “Can you tell me a bit more? Can you explain? Can you give an example? – were posed by the two interviewers. After each interview the interviewer summarized and asked the parents if it was understood correctly. Time and place of each interview was determined in consultation with the parents. Seventeen interviews took place in the homes of the families and one at the parent’s place of work according to the parent’s preferences. Each interview lasted for between 45 and 90 minutes and contained valuable and rich descriptions of each parents’ experiences. The interviews contained lots of data and informational redundancy was reached. All interviews were transcribed verbatim by the first author (two interviews) and by a secretary.

**Table 2:** Interview areas

	
Focus on education/training	- how was it?
Person / people you remember from the time in the hospital?	- for any reason
The understanding of the implications of the diabetes	- how did you know about the disease?
	- difficult to understand?
	- support to get an understanding of the disease
Approaches and preparedness	- approach to learning to take blood, measure blood glucose, prepare the "pen" and give insulin injections
	- preparedness to care for your child with diabetes during and after leave and discharge
Decision making	- about what is to be learned
	- on leave (how was it? /what happened after the leave from the hospital?)
	- own ability to influence the care of the child, teaching content and design
	- on discharge
Something that could have been done differently?	- most important / most useful of what happened during the hospital stay

### Theoretical framework

The theoretical framework is based on the philosophical theory *The Logic of Care* by Annemarie Mol [16] from the Netherlands. Mol argues that good care has little to do with “patient choice” and concludes that good care is something that grows out of collaborative and continuing attempts to attune knowledge and technologies to diseased bodies and complex lives. The standard of individual choice in health care, as it is advocated in health care laws in Sweden and Europe today, was to get away from the previously dominant view that when a patient meets a doctor, the doctor observes, investigates and prescribes different tests without listening to the patient. In order to be able to make choices, that may have a critical effect on the patients’ lives when being affected by a disease, they need to be heard and respected as subjects. Mol [16] argues that if a person has just been diagnosed with, for example diabetes, it is most likely that the person is scared and confused and in that situation would like the health care staff to make the choices for him/her. In this situation the patient needs to be involved in the practical measures that the therapy includes. This is a world infused with what Mol calls *the logic of choice*. In care practices patients are not passive i.e. patients are active, not as subjects of choice but as the subjects of all kinds of activities.

The logic of care concentrates on the kind of activities within which the patient is engaged. In order to help create a good life for the patients in spite of their illness, the professionals need to be open towards them and to share with them the crucial and substantive issues such as, for example, learning. How to live well and learning about what can be fatal depending on the disease involved [16]. The theory includes terms that will guide how the logic of care and the logic of choice are to be applied in practice, i.e. patientism, doctoring, shared doctoring, activity, sensitivity and individuation, described in Table 3.

**Table 3:** Terms used in ”The Logic of Care” and the application to T1DM

	
Patientism	Establishing living with a disease, rather than normality as the standard. The patient has to be aware of what is happening in the body and to respond and adapt to what is happening. Patientism means that the professionals motivate the patients to recognize their body’s signals for wellbeing and malaise. The professionals are admitted into every individual patient’s lifestyle and values and are aware of the sick body’s reactions i.e. the professionals and patients jointly explore ways to achieve a good life despite a fragile body.
Doctoring	The professionals interact with the patient but it is the patient who must control the teaching of diabetes and how the treatment will be implemented. What happens in the body when the body is affected by diabetes is something that the patient must understand as well as how diabetes is generally treated. For example, diabetes patients have to be able to inject their own insulin, measure their own blood glucose level, and count the carbohydrates they eat as well as calibrate their exercise. The professionals show the patient a great commitment by paying attention to the patient’s emotional reactions. This requires endurance and understanding of the difficulties that the patients may feel that they have in daily life with diabetes. Doctoring means that the teaching is fully adapted to the patient’s needs.
Shared doctoring	The professionals adapt their knowledge to the patient’s life and both parts are open and honest in the communication. All persons involved in the care must respect each other's experience of the disease, while being committed and creative as well as careful in their explorations. That requires that all those concerned i.e. members of the diabetes team and the patient, are taking each other's contributions seriously, and they simultaneously adapt to what the body, blood glucose measurements, diet and other relevant information shows.
Activity	This characterizes the patient involvement in the care. The body and the patient are active, i.e. the body is active in that the patient may be thinking that he/she is thirsty and drinks a lot. The doctor asks how much and the patient may respond four litres a day and the doctor interprets it as the symptoms of diabetes. The patient is active by giving blood and urine to be tested. The patient cooperates with the diabetes team (if he/she is not in a diabetes coma) by observing what is done, by asking, listening and performing the care.
Sensitivity	The patients must train their own body and sensitivity so they can actively balance the energy they need with the amount of insulin they inject. Such intro-sensing is an intriguing skill that may be trained. The sensitivity is about making the patient aware of, and helping her/him understand, how she/he can appreciate the blood glucose value by learning how the body works at low blood glucose such as with dizziness or irritation. It also about finding out how to measure so that technologies, habits, etc. are adjusted to the patient’s life.
Individuation	A person with diabetes must learn to become a special person. In the logic of care this is about being an ordinary person yet having the personal courage to be different in this new situation. The PDSN has an important role in supporting the diabetic person, and encourages and praises when the person stands up for her/his own life and the dietician may be the one who gives support to give up a diet that is not necessary. In time the patients gradually gets used to their new situation by taking the insulin without hiding, by abstaining from desserts with a good feeling etc. It is about choosing to participate in social life despite the disease.

### Analysis

In a first step the transcripts were read by all three authors. In a second step all text that seemed to concern information and/or teaching about T1DM, as well as about any emotional reactions, was highlighted to make sure that all possible occurrences of the phenomenon, i.e. the educational process when a child in the family is newly diagnosed with T1DM, were captured [17]. The third step in the analysis included each interview being divided into units of meaning, i.e. sentences that were related in meaning were grouped together in the same meaning unit [14]. In the fourth step each meaning unit was condensed to shorten the sentences but still save the core [14]. Then, in a fifth step, each condensed meaning unit was labelled according to specific codes; in this study, according to the theoretical framework, the terms patientism, doctoring, shared doctoring, activity, sensitivity and individuation are used [16]. The encoded texts from each interview were thereafter placed under each other and read repeatedly to make sure that the content was true to the intentions of the code content. After this the text was summarized under each code and all interviews were read once again in order to confirm that all text that was relevant for the purpose was included. The different steps in the analysis were first performed separately by the first and the third author and thereafter discussed and reflected upon until consensus was reached. Secondly, the second author, a paediatric nurse with 30 years’ experience of working with children and their families reviewed the preliminary result. Finally, all three authors reflected upon and discussed the results by going back and forth between the interview text and the predetermined codes until consensus was obtained. Before consensus some codes were revised, and some meaning units that contained too little or inadequate information was excluded. The results are illustrated by quotations from the original interview text. The quotations are marked with numbers to show the interview from which the quotation derives.

## Results

The results are described according to Logic of Care: patientism, doctoring, shared doctoring, activity, sensitivity and individuation.

### Patientism

The parents usually found themselves in a state of shock and had had practically no previous experience of T1DM when the child was diagnosed with diabetes. A diabetes team consisting of a DSP, a PDSN, a dietician, a social worker and psychologist often introduced themselves during the family's first days in the hospital and gave the family a planning timetable for the hospital stay. This rapid initiation of therapy and making of appointments with the diabetes team was experienced by the family as confusing but, at the same time, as providing a sense of security. The parents were on sick leave for about a month after diagnosis. Both parents were recommended to stay at the hospital with their child during the planned admission, i.e. for 10–14 days depending on the child’s age and status. The parents described that they usually both stayed at the hospital in the daytime, but often only one parent stayed overnight.

When a preschool child protested against taking blood glucose measurements or insulin injections the parents reported that the professionals tried to cooperate with the child in various ways. Sometimes, the child did not accept, but the professional then set a limit and said that it had to be done and proceeded to inject the child.

“Because it was a period when she refused to [go with the insulin injections] …They tried to coax for a while but then stopped, and it was good because I learned that this is the line as well, now you just do it” (2, mother).

Some families had felt that at times the professionals were not responsive enough to the child’s opposition towards insulin injections. The child would communicate via its parents and the professionals gave in after a while and left the room whereupon the child did not want to speak or respond to the professionals any more. In this situation one mother took a decision together with the child to manage the injection by themselves in spite of having no training in giving injections but having watched the professional’s procedure. The mother and the child did it and all of the times afterwards. Both the family and the professionals were satisfied with the outcome.

### Doctoring

The parents experienced that they had received a wonderful reception and excellent care when entering the paediatric hospital. The professionals took care of the child directly and the parents saw them as being knowledgeable and calm. Being informed about the good treatment that exists today and about the parents not having been able to prevent their child from getting T1DM was very important as they immediately got feelings of guilt and wondered if the onset would have been possible to avoid.

“The DSP and the PDSN reassured us [parent and child] by saying that we will know everything before going home, which had a calming effect” (3, mother).

The parents had the impression, when encountering the DSP and PDSN that they had a great deal of knowledge and extensive experience of diabetes care as well as a strong commitment to teaching the parents to be able to take care of their child’s body affected with diabetes. At first the DSP lectured on diabetes and its treatment and after that the PDSN took over with a similar content, but in a more practical way. The DSP and the PDSN overlapped when it came to knowledge about T1DM, which was appreciated by the families, as it became a form of rehearsal. The families who did not receive this commitment and confirmation experienced that they never understood the disease during the period of hospitalisation.

The parents felt that the DSP and the PDSN had taught them about what had happened in the child's body and what the body needed in order to be able to function in the future. The teaching continued step by step about how diet, activity and insulin affected the child's body. Both children and parents were recommended to be present, but sometimes the child, depending on his/her age, went to play-therapy. When the child was present, the professionals turned to the child with the teaching and also emphasised, with eye contact, the seriousness of having T1DM. This presentation was experienced by the parents as a support in getting the teenagers, in particular, to understand the seriousness of the situation.

” … I think she realized pretty quickly at the first appointment with the doctor when he explained to her that this is a dangerous disease… she understood that this [the disease] was nothing that she lost the next week but would have to live with it” (8, mother).

The education was experienced as being intense. Parents felt like they were being made a cram of knowledge, especially when the child was not present. However, the parents accepted this as they wanted to learn as much as possible about the disease. A few parents felt that they never really became acquainted with the diabetes team and found no structure in the learning during the hospitalisation. They became frustrated because they felt they did not understand the diabetes or the management of it at all. They were full of questions but could not formulate them. Many experienced the learning as being about what to do but not about why it should be done, which lead to their feeling of overprotecting the child after discharge. It was not until three months after discharge, when the family had an appointment with the diabetes team, that they gained a fuller understanding of the diabetes and the care.

Some parents expressed that they wished the professionals had given more active advice on how to live with diabetes at home. All the parents were struggling to be good parents; both in terms of managing the child's diabetes but also in terms of ensuring that the child's life would continue to be as normal as possible despite the diabetes.

“Sometimes it may feel easier and safer to say no [relating to food and compromise on time], but it’s all about the child’s life not about our [the parents] life” (7, mother).

### Shared doctoring

The professionals were perceived as having a lot of patience and an open-minded attitude towards the parents when they found it difficult to absorb and understand the disease and care. Parents highlighted that the DSP often drew cartoons that made it easier to understand the teaching. All the families had received brochures and a book about T1DM to read on their own. In spite of intense days there was, nonetheless, time for reflection and discussion for the parents among themselves.

Some families found calculating the insulin dose difficult but they were then offered a template so as to facilitate in making the decision about how much insulin was needed in relation to the blood glucose value. Blood glucose was measured before and after every meal and at night. The same adherence to diet and insulin doses as during the hospitalisation was followed during the one to three months after discharge. Gradually the professionals left the issue of how much insulin the child should have in relation to blood glucose level and diet. The parents saw this as a positive signal allowing the family to gradually become more independent in their management of diabetes.

Great emphasis was placed on the symptoms of hypoglycaemia but teaching about what to do in the event of hyperglycaemia was limited. The parents expressed that problems with hyperglycaemia often arose in the evenings or during the night after discharge due to a lack of knowledge. Many children had sporting activities and the parents appreciated the teaching and advice about what to consider before and after sporting. The appointment with the dietician was based on the child’s diet before diagnosis which was then modified so as to be an appropriate diet for the child after the diagnosis. There was some discrepancy regarding the dietician’s teaching efforts but, on the whole, the parents appreciated the dietetic advice.

“…Dieticians …they are so far from reality and are so careless with food. They have their learning as well, eh … But it’s good, you should aim high” (4, father).

Sometimes the children could choose a blood glucose meter and insulin pen while others only had the opportunity of choosing the colour of the blood glucose meter. Some of the parents thought it was safest to choose the same blood glucose meter and pen that had been used at the hospital. The parents reported that the choice of insulin was not discussed with the parents.

The parents expressed having felt that a spirit of compassion and caring permeated the entire hospital stay. It was encouraging to both of the parents that they were on sick leave and were at the hospital during daytime so they could support each other both in the medical caring and in their sorrow. Parents and their children very much appreciated the play-therapy and the hospital school. It was a way of forgetting the disease and the misery they felt, at least for a while.

The parents reported that after having been in hospital for 10 to 14 days they longed to go home; even if they did not feel they had the care completely under control they felt ready for discharge. Parents were informed that they could phone the DSP or PDSN if there were any problems.

### Activity

Prior to diagnosis, the majority of parents had presented non-specific complaints such as enuresis or weight loss in the child. Immediately the doctor suspected that the child had T1DM, and referred the family to the nearest paediatric hospital. At the hospital the diagnosis was confirmed with new blood samples and the first information about the disease was given.

According to the parents, the professionals at the ward and the PDSN taught them and their children to take care of the practical stages such as taking blood glucose measurements as well as giving and/or taking insulin injections. To begin with, the professionals showed them how to do it and the child and their parents watched. After the family had practiced giving injections using an orange, the parents pricked themselves in the finger to take blood glucose measurements and injected sodium (NaCl) into their stomachs. Some parents pointed out that the professionals never asked if they were afraid of injections, they just assumed that the parents would be able to inject themselves.

“Today it’s dad’s turn to try and inject himself in the stomach [said the PDSN]… Well, you couldn’t back down so it was just to go ahead and do it and it was all right”(5, father).

The parents reported that the professionals gave the injection to the children until such time as they either felt ready to inject themselves or their parents were ready to inject them after some boosting and encouragement. The children either injected themselves immediately or it could take days or weeks until they felt ready to inject the insulin by themselves. In the case of the preschool children it was often the parents who took the blood glucose measurements and gave the insulin injection to the child.

The parents told of how, before discharge, parents and children over 10 years of age had a kind of examination of their acquired knowledge and skills, answering a questionnaire and discussing the answers with the PDSN. Another way of evaluating was when the PDSN asked the family what they planned to do the next few days after discharge and how they thought they would act in various situations that might arise.

### Sensitivity

When young teenagers objected to taking their insulin injection the professionals would try to talk them to terms by asking: “What happens in your body if you don’t receive insulin?” This treatment was appreciated by the parents as a good approach and a great way of dealing with young people.

The teaching included information that the blood glucose levels should correspond to certain values, even though some parents noticed that their child got hypoglycaemia at other values.

“Hypoglycaemia did not come until … he can be low down on the three … without having a hypo … he has even made measurements for three with no feeling of hypo. It took some time for him to believe it was a difference between being low and having hypo …” (6, mother).

During the first few days the families felt restricted to the ward. Gradually, they were encouraged to stay in the hospital environment for brief periods. In a dialogue with the diabetes team the appropriate time for going on a short leave was determined. For some families, this might take place at an early stage due to some kind of celebration, such as breaking-up day or Midsummer. The parents reported that such short leaves were endorsed by the DSP and the PDSN and were well planned together with the family.

The diagnosis came as a shock and the parents experienced that they were saddened by the news that their child had got a chronic disease. At the hospital, the parents described themselves as living in the moment because they wanted to learn to take care of the sick child's body but, at the same time, they felt sad inside. They had to keep up a “brave” face for their children and they only surrendered to crying when they were alone. The parents expressed that it was disappointing that there was no time for grief and shock. The social worker and psychologist included in the diabetes team had introduced themselves to most of the families but no specific appointments were planned except that they all met the social worker for a discussion of social benefits regarding the child’s chronic illness. Some parents did not feel ready to talk with a psychologist, while others lacked the opportunity.

“I felt like we put our problems in their hands and they took care of them in a nice way… it’s not just facts and figures but there’s also a concern” (6, father).

### Individuation

Parents were quick to realise that the care of the child's diabetes required a solid structure in their daily lives but the willingness to learn about how the child's body functioned was immediately apparent.

“I asked the PDSN about how well I have to manage her disease. Must it be to 120% or if it was okay with 80% sometimes. The PDSN replied that it was okay with 80% sometimes. It was a relief” (7, mother).

The parents described how the dietician took the family to a grocery store and the family told the dietician about what kinds of food they used to buy. The dietician then gave them alternatives they could choose from and guided them as to what to think about when shopping. Several of the children had previously drunk a lot of milk and had eaten white bread but, on the whole, the change of diet was uncomplicated when the dietician and the family discussed, and found, alternatives.

The PDSN had informed the teachers and pupils at the child's preschool or school about T1DM and what it means to have this disease. Furthermore, she informed them about how the teachers and pupils could help the child, both in everyday life and in the event of an emergency such as hypoglycaemia. The parents also said that often even relatives were given basic information about T1DM.

“It is important that those who take care of her in school know what to do. She can inject the insulin by herself, but you have to make sure she puts the pen on the correct insulin value… She's only seven years old…” (8, father).

The parents reported that they brought the routines they had learned at the hospital back home. It was important to adhere to the same times and procedures with regards to diet, insulin and different activities. All routines had an influence on family life and made a big change in their social situation. The parents felt sad that life could not be the same as before; they could no longer do things spontaneously in their family since everything would have to be planned in advance.

“It is very focused on times … when we had guests in the evening and they called and said they will be 30 minutes late, you had to say it was okay, but it meant that our daughter had to eat before the guests arrived … so it affects the social side very much” (6, father).

## Discussion

This study was conducted to describe parents’ perceptions of the educational process when their child is newly diagnosed with T1DM. The theoretical framework, “The logic of care” by Mol [16] served as a guide for analysing the interviews.

The reason for choosing to apply Mol’s [16] theory to the parents’ perceptions of the educational process when their child is diagnosed with T1DM is that the theory suggests that the professionals must be sensitive to the values of the children and families so that normative facts relate to the families’ lives. The challenge in the educational process, according to the theoretical framework, is that the professionals, the parents and the child must be prepared, at their first meetings together, to share experiences, knowledge and assumptions about the disease and the situation that the family is in. The professionals have to find a common language that everyone feels comfortable with, i.e. the professionals and the children and their families need to support each other. Education is crafted for a more decent way of living with or in reality.

Mol´s theory [16] has been published relatively recently and has, to our knowledge, not been used in research so far. We do not know if it is practically applicable in the care of children newly diagnosed with T1DM as it emanates from an adult perspective and outpatient care. Some self-criticism is therefore justified as to how we have interpreted the application of the theory.

The aim of the theory is to avoid unmarked normality and rather seek to contribute to theoretical repertoires that no longer marginalize, but instead face, the disease. It is obvious that the DSP and the PDSN mostly follow the guidelines for diabetes education of children and young people as described by ISPAD [9] at diagnosis. This is in line with the results from a previously conducted interview study with the professionals in the paediatric diabetes team [12]. The guidelines emphasise, just as in Mol’s theory that professionals should learn to incorporate and deliver the education using behavioural approaches which are learner-centred [18]. The educational model for diabetes education is based on the view that the effects are most potent when the education is integrated into routine care, when parents are involved, when empowerment and problem-solving principles are involved, when goal setting is performed and when self-efficacy is promoted [19]. The five-step empowerment model which has been shown to be effective and evidence-based in previous studies [18,20,21] emphasising that the patient will be involved in all of the care and reflecting upon how the treatment fits in with the patient’s life is, to a great extent, in concordance with Mol’s theory [16] concerning her emphasis on the patient’s values in the educational process.

Almost all parents felt that they were in good hands and surrendered themselves and their sick children. This can be interpreted as an obstacle to patientism because parents belittle themselves by their strong emotional experience when it turns out that they do not understand anything about the disease. This is not in concordance with the logic of care but is a phenomenon that occurs in paediatrics [22] leading to difficulties for parents who become overpowered by their feelings and thereby no longer have the capability to express their own will. Thus, they may lose their sense of dignity towards themselves and the professionals as well as towards their child. According to the theory, in order to avoid this phenomenon, the professional should start the educational process by asking the parents what they know about diabetes or if they know someone who has diabetes and to have this as a starting point in the teaching. Parents felt strongly that they could rely on the DSP and the PDSN (patientism) for receiving attention and became conscious of their future responsibility for the illness. This is also shown in Wennick and Hallström [23] who interviewed 23 parents to children newly diagnosed with T1DM. In their study, as well as in the present study, most parents were satisfied with the education concerning technical management in the same way as is shown in Challener and Davies [24] who stated that parents appreciate the training of injection techniques, blood testing and diet management i.e. doctoring.

In the logic of care and the logic of choice the professionals should, after teaching the parents what the diabetes does to the child’s body and which medical treatment is necessary, let the parents come to terms with the disease by themselves based on the activities within which the parents and the child are invited to participate [16]. However, in our study the parents’ own reflections, i.e. sensitivity, were not always asked for, i.e. values, on how they planned to cope with everyday life with diabetes. On the other hand, maybe the parents did not ask for time for reflection together with the professionals and therefore the professionals felt that there was no need to address this issue. If the theory works, the parents are supposed to bring to the fore their concerns of their everyday life in an open and honest manner and, together with the professionals find a better lifestyle for the family, i.e. shared doctoring. However, as experienced by some parents, it could take time for them to both become aware of the concerns and to be able to formulate them. Sometimes it took several months and it was not until the meeting at the out-patient clinic that an honest relationship was established.

Although the parents in our study do not mention that they seek control in their lives by following the hospital routines we can imagine that they are struggling for control. A life with diabetes can never lead to full control because life changes all the time and the most important prerequisite for living a good life with diabetes is to listen to the signals from the child’s sick body [16]. During the educational process it does not seem to be the child’s body signals that are in focus. For example, all parents get the same target of low and high blood sugar levels irrespective of at what level the child gets hypoglycaemia. Based on the theory, Mol insists that doctoring is all about making a tailor-made assessment for each individual; in this case it would be deciding the blood sugar value that is tailor-made for each child according to the signals of the body. The parents’ own participation in the various training elements highlighted by Mol [16] was not as could be expected, instead the parents experienced that there was a plan to be followed. Professionals ticked off the various points as they were performed on the checklist, for example the handing over to the parents of leaflets and a book, without discussing them. The parents reported that neither the day nor time intended for practicing to give injections is discussed but instead decided by the professionals.

Being on leave is appreciated as it provides an opportunity for trying out self-care. However, the professionals do not always take the opportunity to ask parents how things have been unless the parents ask for an evaluation. There are good examples of negotiations such as when the dietician accompanies the parents to the grocery and they have discussions based on the parents’ shopping lists, i.e. shared doctoring and individuation. Other examples are when the families may choose blood glucose meters among those that are presented by the professionals, i.e. shared doctoring. The parents also spontaneously take the opportunity to assume their own values to solve problems, for example, when the child refuses to be given insulin injections and the mother and the child manage it without interference of the professionals, i.e. patientism.

The parents felt sorrow when their child was diagnosed with T1DM, but during the hospitalisation there was no time for grief. Thus, the parents learned about the disease and kept their sadness inside. According to Mol [16] the sorrow has to be put aside in order to be able to focus on how to learn about the sick body. However, other studies suggest that the professionals not only assess the child’s wellbeing but also look for symptoms of anxiety among parents especially during the first months after diagnosis [8,25].

It is found that parents coming home with children newly diagnosed with T1DM have difficulties in adapting the regime and the enhanced need to stay in control to their ordinary life [23,26,27]. In our study the families had similar experiences and they continued using the rules they had learned at hospital when they got home without adapting the rules to the family’s lifestyle. This may, according to the theory, mean that the family members themselves have made this choice, possibly without understanding how much it affects the family, and therefore feel a limitation in that situation.

Different steps were taken to secure trustworthiness [28]. To obtain variation in participants all parents to children diagnosed with T1DM were consecutively asked to participate and 10 out of 14 available families accepted to participate. The four families that decided to not participate in the study may have different experiences and this is important to take in consideration when discussing the results and if the results are transferred to another context. There was a risk that an inconvenient time and setting might affect the content of the interview [29]. Thus, all of the interviews were conducted at the families' convenience and usually in their homes, as we hoped that this would help them feel free and comfortable. In the interviews parents were first asked to narrate their own unique experience about the care during hospitalisation. If some areas needed to be explored more, questions and follow-up questions were asked in accordance with the interview guide [30]. To further increase credibility the parents were interviewed separately so as to ensure that they would speak of their own individual experiences and also because studies show that there are different opinions about fathers’ responsibilities for children with diabetes [31-33]. However, in our study both parents seemed to be very engaged in the educational process and supported each other. In the analysis process, one researcher who did not take part in the interviews also analysed the interviews to increase the credibility. The majority of parents were Swedes, living in private houses, in both rural and urban areas and most of them were married or cohabiting. Since geographical and cultural constraints and socioeconomic status and chronic disease can affect parenting [34], the results might need to be further discussed before they could be transferred to another context. Qualitative studies produce rich and detailed information on a small number of cases, the value of which lies in the insights that can only be obtained by detailed work [29]. The research process has been described as precisely as possible in order to increase the dependability of the studies. Researchers with different backgrounds independently analysed the data. Interpretations and insights were considered, compared and reflected upon in open dialogues between the researchers and during research seminars with specialized nurses and midwifes. The results were found to be plausible and sensible. Members check by the participants of the study was not done and the credibility in the data collection is dependent upon the close and mutual respect that was established between the interviewers and the parents. To make sure that the researchers captured the parents’ perceptions follow up questions and summaries during the interviews were frequently used. The objectivity of data has been considered by quotations in the results from both mothers and fathers [35].

## Conclusions

The finding is in concordance with “The Logic of Care” in that the professional is felt to be knowledgeable and experienced by the parents, and committed to the family, inspiring trust and confidence in that their child is in good hands (patientism). The theory also stresses that the relationship between parents and professionals is so acquiescent that parents spontaneously talk about their mistakes and problems in everyday life with the professional, which all parents in the study feel comfortable with especially after discharge (shared doctoring).

What is not in compliance with “The Logic of Care” is that the acquisition of knowledge and skills is largely derived from a structured programme that controls when and what is to be learned day by day. The parents are more like passive recipients than active participants involved in the determination of what they are motivated to learn more about from day to day (activity). They are indeed encouraged to ask questions but not to experiment with their knowledge and skills under the guidance of the professionals (doctoring). The parents want to learn as much as possible about the disease and bring all the routines learned at the hospital back home which makes the parents’ efforts at being good parents and managing the child’s disease, (individuation) difficult. There is a lack of emphasis on the need for the parent and child to primarily be alert to, and recover sensitivity towards, the child's body signals when it is unwell. The family’s grief was set aside during the intense educational process and it is important for professionals to be aware of family members’ feelings so they can be attentive to those in need of support (sensitivity).

In order to optimize the educational process for families with children newly diagnosed with T1DM an increased focus on the families’ perceptions might be helpful in that this could lead to further revelations of the educational process thus making it more understandable for the family members involved. However, more research is needed if one is to know whether or not it is beneficial to use the theory of Mol [16] in a paediatric context.

## Competing interests

The authors declare that they have no competing interests.

## Authors’ contributions

LJ, IH and AL were responsible for the conception and design of the study and the drafting of the manuscript. LJ and AL performed the data collection and the data analysis discussing the results with IH throughout the process. IH obtained funding and LJ and IH provided administrative support. All the authors read and approved the final manuscript.

## Pre-publication history

The pre-publication history for this paper can be accessed here:

http://www.biomedcentral.com/1471-2431/12/165/prepub
